# Comparative Analysis of Proximal Tubule Cell Sources for In Vitro Studies of Renal Proximal Tubule Toxicity

**DOI:** 10.3390/biomedicines13030563

**Published:** 2025-02-24

**Authors:** Courtney Sakolish, Han-Hsuan D. Tsai, Hsing-Chieh Lin, Piyush Bajaj, Remi Villenave, Stephen S. Ferguson, Jason P. Stanko, Richard A. Becker, Philip Hewitt, Weihsueh A. Chiu, Ivan Rusyn

**Affiliations:** 1 Department of Veterinary Physiology and Pharmacology, Texas A&M University, College Station, TX 77843, USA; csakolish@cvm.tamu.edu (C.S.); hanhsuan.tsai@tamu.edu (H.-H.D.T.); hclin@tamu.edu (H.-C.L.); wchiu@tamu.edu (W.A.C.); 2 Global Investigative Toxicology, Preclinical Safety, Sanofi, Cambridge, MA 02141, USA; piyush.bajaj@sanofi.com; 3 Roche Pharma Research and Early Development, Roche Innovation Center Basel, F. Hoffmann-La Roche Ltd., 4070 Basel, Switzerland; remi.villenave@roche.com; 4 Division of the National Toxicology Program, National Institute of Environmental Health Sciences, Research Triangle Park, NC 27709, USA; stephen.ferguson@nih.gov (S.S.F.); jason.stanko@nih.gov (J.P.S.); 5 American Chemistry Council, Washington, DC 20002, USA; rick_becker@americanchemistry.com; 6 Chemical and Preclinical Safety, Merck Healthcare KGaA, 64293 Darmstadt, Germany; philip.hewitt@merckgroup.com

**Keywords:** kidney toxicity, in vitro models, model-omics

## Abstract

**Background/Objectives:** The kidneys are essential for eliminating drugs and chemicals from the human body and renal epithelial cells are particularly vulnerable to damage caused by xenobiotics and their metabolites. Drug-induced kidney toxicity is a major cause of drug attrition during preclinical and clinical development and the ability to predict renal toxicity remains a pressing challenge, necessitating more predictive in vitro models. However, the abundance of commercially available renal proximal tubule epithelial cell (RPTEC) sources complicates the selection of the most predictive cell types. **Methods:** This study compared a wide range of RPTEC sources, including primary cells (Lonza) and various RPTEC lines from different vendors, such as ciPTECs (Cell4Pharma), TERT1/RPTECs (ATCC), and HEK293 (GenoMembrane), including OAT1-overexpressing variants. HepG2 cells were included for a comparison of organ specificity. The different cells were cultured in 96- or 384-well plates and exposed to 12 drugs for 72 h at a concentration yielding a response (0.3–300 µM) to evaluate their ability to predict clinical outcomes. The CellTiterGlo^®^ assay was used to measure cell viability, and transcriptome data from unexposed cells was analyzed using the TempO-seq^®^ S1500+ platform. **Results:** Gene expression data showed that the primary kidney cells most closely matched the transcriptome of the human kidney medulla, followed by the TERT1 and ciPTEC lines, with the HEK lines showing the lowest similarity. The RPTEC sources showed clustering by cell type, with OAT1 overexpression driving changes in metabolic, detoxification, and immune pathways, especially in TERT1 cells. Cell viability data were used to determine points of departure (PODs) which were compared to human serum Cmax values to assess safety margins. The TERT1 and ciPTEC RPTEC lines demonstrated the highest predictive performance for nephrotoxicity, with OAT1 overexpression significantly enhancing sensitivity, accuracy, and overall predictive power (MCC scores: 0.764 and 0.667, respectively). In contrast, HepG2 cells showed the lowest performance across all metrics, highlighting the critical role of cell type and transporter expression in nephrotoxicity prediction. **Conclusions:** This study highlights important differences among RPTEC sources and their utility in drug safety studies of the renal proximal tubule. We show that while improved cell options for renal proximal tubule are needed, *OAT1*-overexpressing RPTECs are a superior model to the background cell type.

## 1. Introduction

The kidney plays a key role in drug and chemical toxicokinetics because it is responsible for clearance through glomerular filtration, tubular secretion, metabolism, and reabsorption [1,2]. The kidney is also a vital organ that maintains overall blood composition, pH balance, and electrolyte levels; it filters 150–180 L of blood daily to produce 1–2 L of urine [3,4]. Given the importance of the kidney in drug toxicokinetics, investigational drug candidates are characterized by renal transport and potential nephrotoxicity. Indeed, given that the kidneys are frequently the primary site for the elimination of various compounds, and with 32% of the top 200 prescribed drugs in 2010 undergoing renal elimination, nephrotoxicity is a common concern [5]. Drug-induced toxicity is an important cause of acute kidney injury; between 20 and 60% of acute kidney toxicity cases have been attributed to nephrotoxic drugs, especially in cohorts of patients with co-morbidities and impaired kidney function [6,7]. Kidney toxicity contributes to approximately 9% of drug termination in the clinical phase [8] and is often identified late in drug development due to the inadequate sensitivity of preclinical models and the limitations of the human blood and urine biomarkers of nephrotoxicity [9,10,11]. The development of improved pre-clinical models for experimental and computational prediction of drug-related nephrotoxicity has been a subject of active research [1,12,13,14].

While the entire nephron is responsible for the physiological processes regulated by the kidneys, the proximal tubule plays an especially critical role in the disposition of various xenobiotics, it is also a common site for toxicity because of the abundance of uptake and efflux transporters and drug-metabolizing enzymes [2,15]. A wide range of renal proximal tubule cells (RPTECs) are available to study the effects of drugs and xenobiotics on this critical region in the nephron [1,14,16]. In addition, RPTECs can be cultured in a variety of ways, from monolayers in multi-well plates to Transwells^TM^ and various organ-on-chip configurations [17,18,19,20]. Because of the importance of renal transporters, several human immortalized RPTEC models that overexpress various transporter proteins have been established from both renal cell carcinomas and non-diseased tissues [16]. Several cell lines, such as HEK293 [21], RPTEC/TERT1 [22], and ciPTEC [23], have been particularly popular choices for studies of drug-associated renal toxicity because they were derived from normal human kidney tissues. Stably overexpressing variants of these cell types are available for several key transporter genes to improve the physiological and toxicological relevance of these human cell-based models [24,25,26].

Limited access to freshly isolated RPTECs highlights the need to explore alternative cell sources that can deliver high sensitivity and specificity in vitro. Consequently, further research is needed to evaluate the strengths and weaknesses of various options for cell sources and culture modalities. A number of recent reports presented data on comparative analyses of panels (typically several dozen) of nephrotoxic compounds in several cell types, most often using freshly isolated RPTECs, or immortalized RPTEC lines and their organic anion transporter 1 (OAT1/SLC22A6)-overexpressing variants [27,28,29,30,31,32,33,34]. These studies were conducted in traditional multi-well plates and used a variety of approaches to phenotype the effects of drugs, from the determination of cell viability to gene expression and other molecular markers. The accuracy of the nephrotoxicity predictions reported in these studies was as high as 80%, and generally greater when combinations of biomarkers were used to make the predictions. Still, no study has focused on cross-cell comparisons—a practical question faced by many end-users who are concerned about selecting a particular model for long-term use for drug candidate screening. Therefore, this study focused on the evaluation of different cell sources that have been most frequently used—these include both immortalized RPTEC lines and their -*OAT1* overexpressing variants, freshly isolated RPTECs, and a liver-derived cell line, HepG2. Here, we provide a comparison of the gene expression data across RPTEC lines and demonstrate their responses to a compendium of nephrotoxic compounds. The predictivity of these cell types and vitro-derived margin of safety (calculated using both free fraction and nominal concentrations) values were calculated.

## 2. Materials and Methods

### 2.1. Chemicals

The drugs used in this study are listed in Table 1. Cisplatin (#1134357), Carboplatin (#C2538), Dapagliflozin (#SML2804), Gentamicin (#G1397), Streptomycin (#1623003), Acarbose (#A8980), Ribavirin (#R9644), Telithromycin (#SML2162), Tenofovir (#SML1795), Rifampin (#R7382), and Adefovir (#SML0240) were purchased from Sigma-Aldrich (St. Louis, MO, USA). Canagliflozin (#A11100) was purchased from AdooQ Bioscience (Irvine, CA, USA). All compounds were dissolved in DMSO (#D8418, Sigma-Aldrich) at a 200× final concentration, to achieve a 0.5% DMSO vehicle concentration in the treatment media.

### 2.2. Cells and Culture Medium

In this study, eight RPTEC sources were tested, including two from cryopreserved primary donors and three cell lines: ciPTEC, HEK293, and TERT1 (both the “parent” line and -*OAT1* overexpressing variant were tested for each one of these). We focused our study on *OAT1*-overexpressing cells because they are a commonly used in vitro model for studies of renal clearance and because there is overlap in substrate specificity among renal basolateral and apical transporters [35]. Additionally, HepG2 cells were tested to enable comparisons of drug effects in a non-kidney-derived cell type and one which is commonly used for general cytotoxicity measurements during drug development. ciPTEC-parent and *OAT1*-overexpressing lines were obtained from Cell4Pharma B.V. (Oss, The Netherlands) with an academic license. These cells were cultured in DMEM:F12 (#11039-021; Gibco, Billings, MT, USA) supplemented with ITS (Insulin-Transferrin-sodium Selenite media supplement, I = 5 µg/mL, T = 5 µg/mL, S = 5 ng/mL, #I1884; Sigma-Aldrich), Hydrocortisone (36 ng/mL, #H0135; Sigma-Aldrich), EGF (Epidermal Growth Factor, 10 ng/mL, #E9644; Sigma-Aldrich), 3,3′,5-Triiodo-L-thyronine sodium salt (40 pg/mL, #T5516; Sigma-Aldrich), and 10% FBS (Fetal Bovine Serum, #35-010-CV; Corning Life Sciences, Corning, NY, USA). HEK293 parent and *OAT1*-overexpressing variant RPTECs were obtained from GenoMembrane (#GM1101G, #GM1103G; Yokohama, Japan) and were cultured in DMEM (#11960044, Gibco) supplemented with 10% heat-inactivated FBS (#16140071, Gibco). TERT1-parent and TERT1-OAT1 cells were obtained from ATCC (#CRL-4031, CRL-4031-OAT1; Manassas, VA, USA). These cells were cultured in DMEM:F12 (#30-2006; ATCC) supplemented with the hTERT-immortalized RPTEC growth kit (#ACS-4007; ATCC) and a G418 supplement (final concentration of 0.1 mg/mL; Geneticin G418 Sulfate, Gibco). Cryopreserved primary human RPTECs from 2 human donors (#CC-2553, lots 18TL114340 [abbreviated as Lonza340 herein] and 18TL117405 [abbreviated as Lonza405]) were obtained from Lonza (Basel, Switzerland) and cultured in REGM, Renal Epithelial Cell Growth Medium, (#CC-3190, Lonza) supplemented with the “BulletKit” (#CC-4127, Lonza) that contained FBS (0.5%), human transferrin (10 mg/mL), Hydrocortisone (0.5 mg/mL), Insulin (5 mg/mL), Triiodothyronine (5 × 10^−12^ M), Epinephrine (0.5 mg/mL), epidermal growth factor (10 mg/mL), and antibiotics (100 U/mL penicillin and 100 mg/mL streptomycin). Cryopreserved primary RPTECs were shipped from the vendor at passage 2 and were expanded prior to testing for 2–3 passages. HepG2 cells were obtained from ATCC (#HB-8065) and were cultured in DMEM (#11965092, Gibco) supplemented with 10% FBS (#35-010-CV; Corning Life Sciences) and 1% Penicillin–Streptomycin (#15140122, Gibco).

### 2.3. Cell Seeding, Culture, and Drug Treatments

All cells were cultured at 37 °C and 5% CO_2_ except for ciPTECs which were briefly expanded at 33 °C prior to seeding in plates as recommended by the supplier. Cells were cultured in standard “tissue culture” T-75 flasks (#43064U; Corning) except for the HEK293-parent and -OAT1 lines which were maintained in poly-D-lysine-coated flasks (#658940; Greiner Bio-One, Monroe, NC, USA). On the day of seeding, cell suspensions were added to either 96-well plates (ciPTEC-parent and -OAT1 only; #3904; Corning), or 384-well plates (all other cell sources; #142761; Thermo Scientific, Waltham, MA, USA). The cell culture plates were “tissue culture” grade from the manufacturer and did not require additional coating except for HEK293 cells which required pre-coating the plates with poly-D-lysine. Accordingly, 15 µL of 0.1 mg/mL poly-D-lysine solution (#A-003-E; Sigma-Aldrich) was added to each well of the 384-well plates used for these cells. The plates were incubated for 60 min at room temperature, the solution was removed, and wells were rinsed with sterile water. The plates were allowed to dry for 2 h prior to cell seeding. Cells were seeded at a density of 55,000 cells/cm^2^ into 96- and 384-well plates (17,600 cells/well and 3080 cells/well, respectively). After plating, cells were incubated with daily media changes until confluency was reached as follows: Lonza RPTECs: 5 days; TERT1 lines: 3 days; HEK293 lines: 3 days; HepG2: 4 days. In the case of the ciPTECs lines, a 1 week “maturation” was recommended by the vendor; accordingly, cells were plated and incubated overnight at 33 °C, then cultured at 37 °C for an additional 7 days prior to drug exposure.

On the day of drug treatments, the medium was gently aspirated and a fresh medium was added to the cultures (50 µL for 384-well plates or 100 µL for 96-well plates). An equal volume of 2× solutions of drugs in 1% DMSO was then added to each well. This resulted in final treatment concentrations of 0, 0.3, 1, 3, 10, 30, 100, or 300 µM and 0.5% of DMSO in each well. After drug or vehicle addition, cells were incubated for 72 h before phenotyping.

### 2.4. Cell Viability Evaluation

After the 72 h exposure period, cell viability was evaluated using the ATP-based CellTiter-Glo Luminescent Cell Viability Assay (#G7571; Promega, Madison, WI, USA). Briefly, the CellTiter-Glo solution was prepared following the manufacturer’s instructions. The medium was partially removed from all wells (50 µL for 384-well plates, or 100 µL for 96-well plates was removed), then replaced with an equal volume of the reconstituted assay solution. This solution was triturated using a multichannel pipette to thoroughly mix in each well and assist in cell lysis. Samples were incubated for 10 min at room temperature prior to being transferred to an opaque, white 96-well plate (#3917; Corning). The luminescence from each sample was measured using a microplate reader (SpectraMax iD3; Molecular Devices, San Jose, CA, USA). The resulting luminescence value can be directly correlated with the number of viable cells, and treatment groups were compared to vehicle-treated well within each cell source to derive a % (of vehicle) viability value.

### 2.5. Gene Expression Library Preparation and Sequencing

Cell lysates were collected by aliquoting and pelleting 100,000 cells post-thawing. The pellets were resuspended with 50 µL of TempO-Seq™ Enhanced Lysis Buffer (#SU-01-100, BioSpyder Technologies, Carlsbad, CA, USA) and incubated for 10 min at room temperature to allow for complete cell lysis. The lysate samples were aspirated from each well, transferred into microcentrifuge tubes, and stored at −20 °C until later analysis.

The cell lysates were utilized for the Templated Oligonucleotide Sequencing Assay (TempO-Seq™, BioSpyder Technologies, Carlsbad, CA, USA) as the method for mRNA quantitation [36]. The detailed protocols for TempO-seq, provided by the manufacturer and previously described [37], involved preparing TempO-seq libraries using the human S1500+ targeted transcriptome panel [38], which includes 2982 unique probes for human transcripts. Following the manufacturer’s instructions, the mRNA content of the cell lysates was hybridized by incubating 2 μL of the lysate with 2 μL of hybridization mix as follows: 10 min at 70 °C, a cooling ramp from 70 °C to 45 °C at 49 min, and 1 min at 45 °C. Excess oligonucleotides were then digested in a nuclease-catalyzed reaction for 90 min at 37 °C. The hybridization products were then incubated with DNA ligase for 60 min at 37 °C, followed by heat denaturation of nuclease and ligase at 80 °C for 30 min. A total of 10 μL of each ligation product from each sample was then mixed with an equal volume of PCR amplification mix and amplified in a LightCycler 96 (Roche, Basel, Switzerland) using the manufacturer-recommended settings. Subsequently, 5 μL of the amplified samples was pooled and purified using a commercial PCR clean-up kit (Clontech, Mountain View, CA, USA). The pooled libraries were sequenced using 75 single-end read mode dual index runs with custom primers on a NextSeq 550 sequencer (Illumina, San Diego, CA, USA).

### 2.6. Gene Expression Data Analysis

The raw sequencing reads for each pooled sample, which included 2 lanes of single-end sequencing reads that were 75 base pairs in length, were combined to generate a single FASTQ file for each sample. Next, the Fastp (version 0.21.0) approach [39] was used to process the FASTQ files, trimming the sequencing reads to 50 base pairs with default parameters. These processed FASTQ files served as input for the TempO-Seq data analysis pipeline [36], which utilized the human S1500+ probe manifest file. To facilitate further analyses, the resulting raw count matrix data was aggregated to the gene level if a gene was associated with more than one probe in the TempO-seq assay. The FASTQ files are available from the Gene Expression Omnibus (GEO; accession #GSE268877; reviewer token: ejiduiugvvgpjax).

Before conducting differential gene expression or other transcriptomic analyses, quality control steps were applied to the raw counts as follows: (i) exclusion of samples with uniquely mapped reads lower than 50%, (ii) exclusion of samples with fewer than 200,000 total counts, (iii) assessment of performance in principal component analysis (PCA) using the built-in R function prcomp to visualize sample grouping and any potential outliers, and (iv) removal of genes that were expressed in fewer than 2 counts across 10% of the samples. The PCA did not reveal any sample outliers. The final dataset analyzed herein comprised a total of 23 samples (n = 3 for each cell type, except for TERT1-OAT1, which had 2 replicates) and 2582 unique genes.

Differential expression analysis was performed using the DESeq2 package in R [40]. Significant differentially expressed genes (DEGs) for each pairwise comparison were identified using the DESeq2 results function, specifying the contrast between cell types, and applying a stringent statistical cutoff based on the false discovery rate (q-values < 0.01). The union of DEGs (normalized counts) with absolute log_2_-fold-change values greater than 3 (n = 657) was used for clustering and heatmap visualization of the DEGs between cell types using the R package heatmap. The numbers of up- and down-regulated DEGs comparing the *OAT1*-overexpressing lines to their corresponding parent lines in each cell type were calculated. Pathway analysis was conducted for pairwise comparisons between the *OAT1*-overexpressing lines and the parent lines, as well as for gene clusters derived from the unsupervised heatmap of the union of DEGs across cell types, using the R package *xgr* [41] with the command xEnricherGenes (ontology = “MsigdbC5BP”). The background gene set for these analyses consisted of all genes that passed the low-count removal (n = 2582), as described above.

***Determinations of the Point of Departure (POD) and Margin of Safety***

All cell viability data were normalized to the vehicle control (average of the 0.5% DMSO-treated wells). Normalized data for each condition were then fitted to a curve with a nonlinear logistic function to determine the point of departure (POD) values. For the purposes of this study, IC_10_ was defined as the concentration where the fitted curve exceeded a 10% decrease in viability (90% viability compared to vehicle controls), and EC_50_ as a 50% viability of vehicle control wells. Curve fitting was performed using a script as previously reported [42]. All PODs are included in Table S1. Furthermore, risk characterization was conducted by calculating the margin of safety (MOS; Equations (1) and (2)), utilizing the most sensitive POD across all cell types and comparing it to the maximum concentrations measured in human plasma (C_Max_). Moreover, given the significance of proper in vitro dosimetry in risk characterization, this study also considered the concentrations of free chemicals in the media of the in vitro system and in human plasma. Two distinct types of MOS were calculated as follows:

(1) $${MOS}_{Nominal}=\frac{{POD}_{Nominal}}{C_{Max,total}}$$

(2) $${MOS}_{Free}=\frac{{POD}_{Free}}{C_{Max,free}}=\frac{{POD}_{Nominal}\times f_{ub,media}}{C_{Max,total}\times f_{ub,plasma}}$$

where POD_Nominal_ denotes the nominal POD derived from the dose–response profiles, C_Max,total_ signifies the maximal concentration measured in the plasma, POD_Free_ represents the free POD adjusted by the fraction unbound in the tested media (f_ub,media_), and C_Max,free_ denotes the maximal free concentration in plasma adjusted by the fraction unbound in plasma (f_ub,plasma_). Chemical-specific C_Max,total_ values were gathered through a literature review, and the respective ranges are summarized in Table 1. Values for f_ub,plasma_ for each chemical were primarily obtained from previous publications [43] or sourced from the CompTox Dashboard (https://comptox.epa.gov/dashboard/, accessed on 17 February 2025) (Table S2). To calculate f_ub,media_, the in vitro mass balance model (IVMBM) [44] was employed. Chemical-related parameters such as melting point, solubility, and partition coefficients (K_ow_, K_aw_, K_oa_), necessary for IVMBM, were acquired from the Exposure And Safety Estimation (EAS-E) Suite platform (https://arnotresearch.com/eas-e-suite/, accessed on 17 February 2025) or from publications (Table S3). Log K_aw_ values for Cisplatin, Carboplatin, Dapagliflozin, and Telithromycin were not found in the literature, or on the EAS-E Suite, so a default value of −30 was assumed, similar to other non-volatile compounds. System-related parameters, including the type of labware and percentage of serum, adhered to the aforementioned information, with detailed parameter tables summarized in Table S4.

***Toxicity Classifications and Cell Ranking***

To assess binary classifications and rank the cells according to their predictive power for nephrotoxicity screening, the compounds were categorized as either kidney toxicity “positives” or “negatives”. This classification was based on the incidence of renal-related adverse events. Compounds with a known history of renal toxicity were assigned to the positive group, while those with a lower nephrotoxic potential and a lack of reported renal adverse outcomes, such as Acarbose and Ribavirin, were classified as negatives (Table 1). Gentamicin and Streptomycin were excluded from the subsequent confusion matrix calculations due to the subtoxic concentrations used in these experiments. In vitro positives and negatives were classified using EC_50_ values as demonstrated above. If an EC_50_ was not derived even at the highest tested concentration (300 µM), the cell determination was classified as in vitro negative. If the EC_50_ was less than 300 µM, the result was classified as an in vitro positive. Sensitivity, specificity, accuracy, and Matthew’s correlation coefficient (MCC) were calculated through confusion matrixes using the following formulas as detailed in [45]:

(3) $$\mathrm{Confusion}\;\mathrm{Matrix},\;M=\left(\begin{array}{cc} TP & FN\\ FP & TN \end{array}\right)$$

True Positive (TP)—correctly predicted positive

False Negative (FN)—positive predicted as negative

True Negative (TN)—correctly predicted negative

False Positive (FP)—negative predicted as positive

(4) $$\mathrm{Sensitivity},\;\mathrm{predictive}\;\mathrm{rate}\;\mathrm{of}\;\mathrm{TP}=\frac{TP}{TP+FN}$$

(5) $$\mathrm{Specificity},\;\mathrm{predictive}\;\mathrm{rate}\;\mathrm{of}\;\mathrm{TN}=\frac{TN}{TN+FP}$$

(6) $$\mathrm{Accuracy},\;\mathrm{predictive}\;\mathrm{rate}\;\mathrm{of}\;\mathrm{total}\;\mathrm{correct}\;\mathrm{classifications}=\frac{TP+TN}{TP+TN+FP+FN}$$

(7) $$\mathrm{MCC},\;\mathrm{summary}\;\mathrm{of}\;\mathrm{TP},\;\mathrm{TN},\;\mathrm{FN},\;\mathrm{and}\;\mathrm{FP}\;\mathrm{predictions}=\frac{TP\times TN-FP\times FN}{\sqrt{\left(TP+FP\right)\times \left(TP+FN\right)\times \left(TN+FP\right)\times (TN+FN)}}$$

These four parameters were used as representative values to rank the overall predictive power of each cell line in accurately identifying renal-positive compounds.

## 3. Results

**Figure 1:**
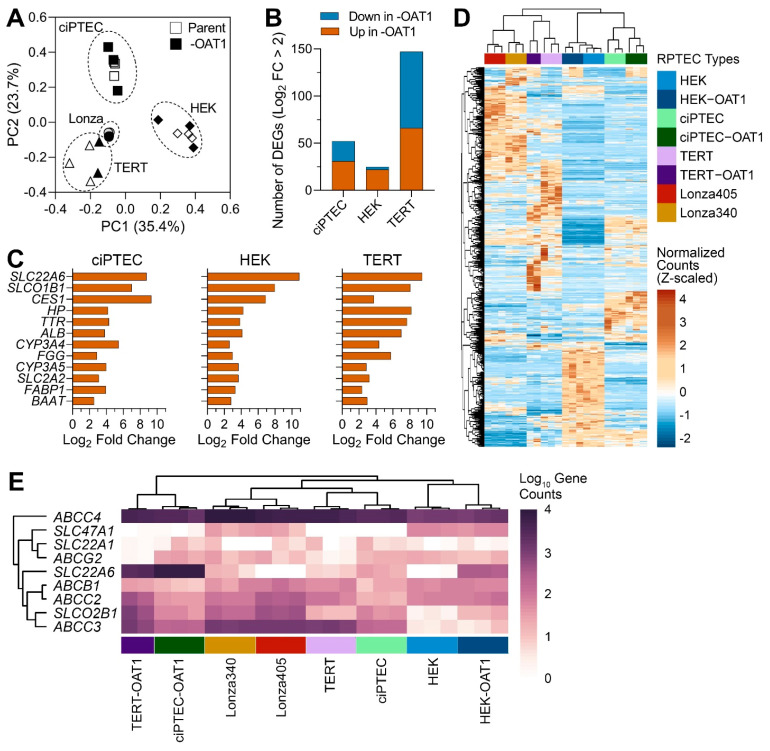
Gene expression analysis of untreated ciPTEC, HEK and TERT1 RPTEC, and their *OAT1*-overexpressing variants, as well as primary RPTECs from two donors (Lonza 340 and 405). (**A**) Principal component analysis of gene expression data. Symbols depict cell lines and variants as indicated in the legend. Cell sources (parent and -OAT1) are depicted in open or solid shapes, respectively. (**B**) Bar plots show the number of genes that were differentially expressed up or down in the -OAT1 variant of each cell source. (**C**) Bar plots show a set of differentially expressed genes that were highly conserved between parent and -OAT1 overexpressing lines. (**D**) An unsupervised (average linkage clustering) heatmap visualizing expression of 657 genes that were differentially expressed between untreated RPTEC cultures from various sources. Colors depict Z-score values for normalized data. Cell sources (vertical-colored bars) are indicated in the legend with technical replicates displayed. (**E**) Unsupervised heat map shows expression levels (in gene counts) among groups for common renal transport genes.

Gene expression profiling has been an invaluable tool for determining the relevance of in vitro models to humans in general [46], and for RPTECs in particular [19,26,27,28,47]. Most studies focus on one cell type or include a limited number of RPTECs when gene expression profiling is performed. While these studies are highly informative, they do not enable a direct comparison of the transcriptional profiles among multiple different cell sources. Therefore, to evaluate the differences between RPTEC sources, we compared gene expression in eight different cells, parent and *OAT1*-overexpressing ciPTEC, RPEC/TERT1 (TERT), and HEK293 (HEK), as well as two primary RPTEC lines (Lonza). Principal component analysis data based on the expression of 2582 genes included in these analyses (Figure 1A) showed that individual samples clustered primarily based on the cell source (parent and *OAT1*-overexpressing cells clustered together in each case); both primary RPTEC lines clustered close to TERT lines. A comparative analysis of the effects of *OAT1* overexpression in each corresponding cell line (Figure 1B) showed that the greatest number of genes affected by such overexpression was found in TERT cells and the smallest in HEK cells. In TERT cells, an equal number of transcripts were higher and lower in *OAT1*-overexpressing cells, but in HEK and ciPTEC most genes were higher in *OAT1*-overexpressing cells. Table 2 shows the pathways and genes differentially expressed among the cell sources. In HEK and ciPTEC cells, genes with a higher expression in *OAT1*-overexpressing cells were associated with biological processes related to metabolism, detoxification, and the transport of organic molecules or ions. In TERT cells, *OAT1* overexpression-associated pathways included immune responses and cell cycle regulation. By contrast, a limited number of biological processes were enriched with higher-expression genes in the parent line compared to *OAT1*-overexpressing ones in both HEK and TERT cells. However, in ciPTEC, genes more highly expressed in the parent line were enriched in broader gene sets, including immune response, nervous system development, and responses to stimuli. The complete lists of DEGs and the associated Gene Ontology Biological Process enrichment results are provided in Files S1 and S2.

Figure 1C shows a set of genes that were both differentially expressed between parent and *OAT1*-overexpressing immortalized cell lines and identical among these three cell types. While it is not surprising that *SLC22A6* (*OAT1*) was among the highest differentially expressed genes, a number of other transporters and metabolism-related genes were shared among the three cell sources. For example, *SLCO1B1* (*OATP1B1*) and *SLC2A2* (GLUT2) are liver transporters with intermediate expression in the proximal tubules in the physiological state. The unsupervised clustering heatmap from the analysis of the differentially expressed genes (657 genes) in untreated RPTEC cultures from various sources (Figure 1D, File S3) showed an overall similarity in the gene expression patterns within each cell type; the differences between cell sources were the primary driver for the clustering of the samples. Secondary grouping within each immortalized cell type was related to *OAT1* expression. Finally, we extracted the data on transporter genes that were examined in this study; the unsupervised heat map shows expression levels (in gene counts) among cell sources (Figure 1E). Only *ABCC4* (*MRP4*) was highly expressed in all cell sources, and to a lesser degree in HEK cells, but largely unaffected by *OAT1* overexpression. Other genes, however, were highly divergent in their expression levels. Except for *SLC47A1* (*MATE-1*), which was moderately highly expressed in HEK cells, almost all other transporters were higher in expression in TERT and ciPTEC.

**Figure 2:**
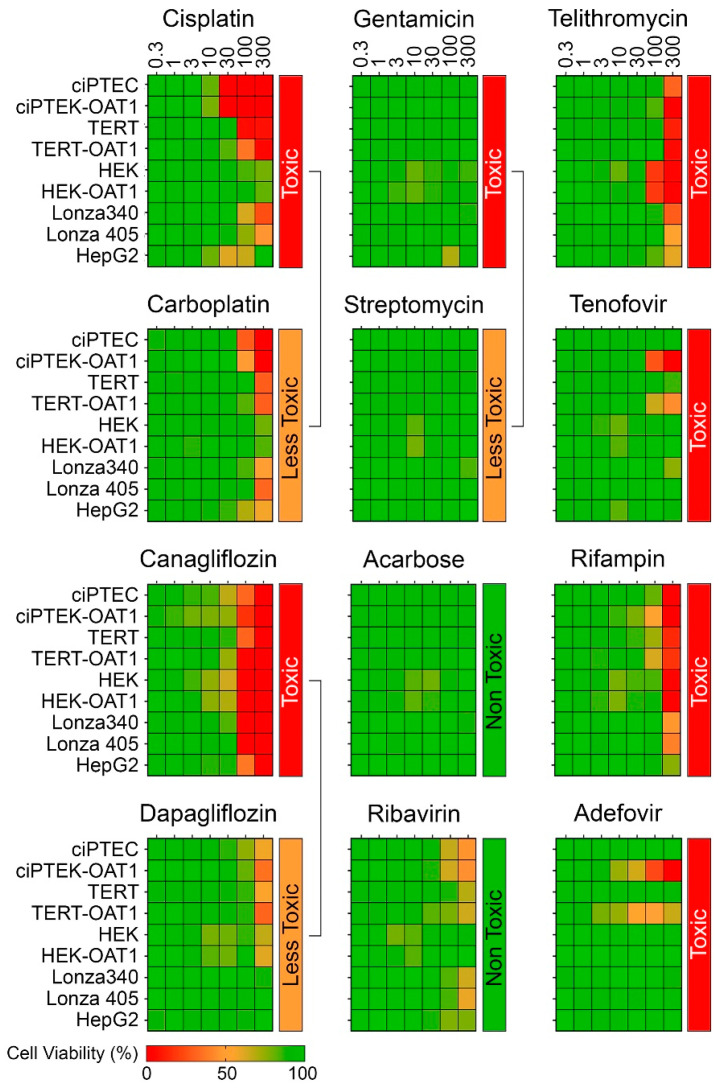
Concentration–response effects in ciPTEC, HEK, and TERT1 RPTECs, and their *OAT1*-overexpressing variants, as well as primary RPTECs (Lonza 340 and 405) and HepG2 cells.

Figure 2 shows dose–response (0.3–300 µM) heat maps for the effects of the tested compounds on cell viability in each RPTEC type and in liver-derived HepG2 cells. A kidney toxicity classification (Table 1) is indicated for each compound, with drugs modulating the same target that vary in nephrotoxicity potential positioned next to each other. Notably, there was significant variability in sensitivity to positive nephrotoxic compounds across the different cell sources, while less toxic or non-toxic compounds showed minimal or no cytotoxic effects across all cells. All ciPTEC and TERT1 cells were comparable in viability post-treatment, except for Telithromycin. *OAT1* overexpression resulted in an enhanced sensitivity to Tenofovir and Adefovir, two antivirals which show uptake via the OAT1 transporter in RPTECs. HepG2 cells showed a consistently lower sensitivity as compared to RPTECs, across all tested compounds. It is of note that no effects were observed for Gentamicin and Streptomycin because they are known to be non-cytotoxic below 300 µM [48,49]. The presence of these antibiotics in cell culture media (at concentrations close to 200 μM) may also be a considerable confounding factor for studies of their nephrotoxicity in vitro [50,51]. Additional experiments with TERT1-parent and -OAT1 RPTECs confirmed that cytotoxicity can be observed at higher concentrations (Figure S1). However, these compounds were excluded from subsequent dose–response analyses due to these considerations.

**Figure 3:**
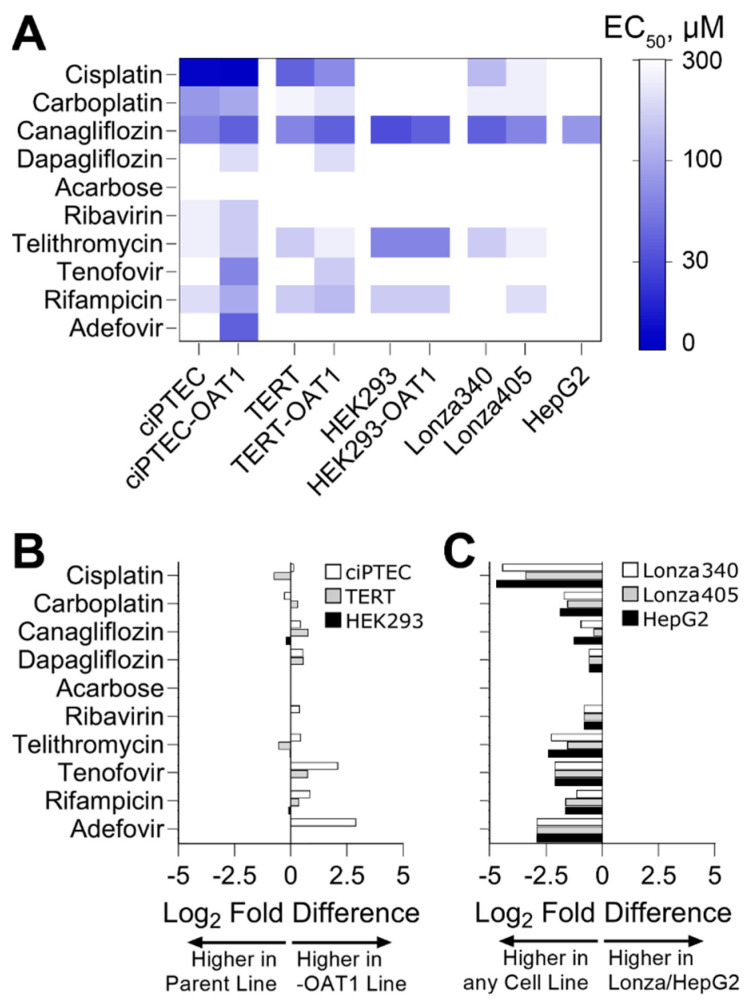
Comparative sensitivity of various cell lines to nephrotoxicants, highlighting EC_50_ values and log2 fold differences in points of departure (PODs) across different RPTEC variants and cell sources. (**A**) Heatmap displaying EC_50_ derived from cell viability data. (**B**) Log2 fold difference between PODs within RPTEC line variants (parent vs. OAT1). If <0, the parent line was more sensitive to changes in viability; if >0, the OAT1 overexpressing line was more sensitive. (**C**) Log2 fold difference between PODs within RPTEC lines and other cell sources (Lonza RPTECs and HepG2). If <0, RPTEC lines were more sensitive to changes in viability; if >0, Lonza or HepG2 were more sensitive.

To go beyond hazard identification, we conducted a dose–response analysis and used points of departure (PODs) values to both enable comparisons of effects for each tested compound among cell sources and to inform risk characterization by comparing PODs to human C_max_ values. Both analyses are routinely used in drug safety evaluation. For PODs, effective concentrations at 50% (EC_50_, Figure 3A) and 10% (IC_10_, Figure S2) were derived. A supervised heatmap of these POD values shows that most RPTEC sources showed similar responses, albeit at different concentrations. HepG2 cells were largely non-sensitive to the effects of the tested compounds, except for Canagliflozin which was cytotoxic in all cells tested. We also compared the sensitivity of *OAT1*-overexpressing RPTECs compared to their parental lines (Figure 3B). For Cisplatin and Carboplatin, the parent cell types for TERT and ciPTEC were more sensitive. For Tenofovir and Adefovir, the *OAT1*-overexpressing cell types of TERT and ciPTEC were more sensitive. For HEK293 cells, the parent and *OAT1*-overexpressing variants were equivalently sensitive. For other compounds, minor differences were observed between cell variants. Another comparison was made between immortalized cell lines (both parent and *OAT1*-overexpressing cells were included) on the one hand, and either primary RPTEC or HepG2 cells on the other hand (Figure 3B). For all tested compounds, except Acarbose that had no effect on any cell type, the immortalized RPTECs were more sensitive than the primary RPTECs or HepG2 cells.

**Figure 4:**
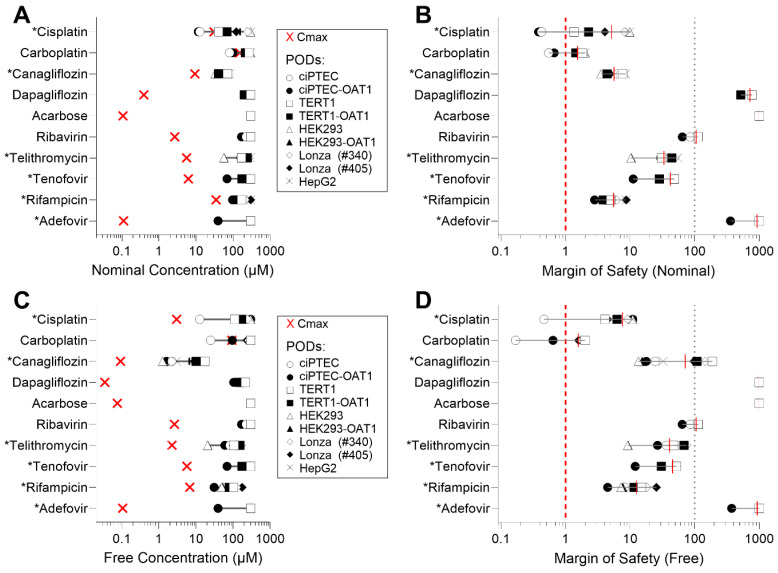
EC_50_ values vs. human C_max_ and margins of safety (MOS) across cell sources. (**A**) Experimental EC_50_ values for each cell type plotted against reported human C_max_. (**B**) MOS calculated using the ratio of human C_max_ to EC_50_. (**C**) EC_50,free_ values (free concentrations in media) plotted against calculated C_max,free_ in human serum, using mass balance modeling. (**D**) MOS calculated using the ratio of C_max,free_ to EC_50,free_, accounting for free concentrations in media and serum. C_max_ values are represented as a red “×” on (**A**,**C**). Asterisk (*) next to the name of a compound indicates its classification as “nephrotoxic”.

Due to the varying sensitivity of different cell types to the tested compounds, we also examined how these in vitro data could be interpreted in terms of their margin of safety (MOS). For this analysis, we compared EC_50_ values to human C_max_, using both nominal (as applied to the cells or reported) and ‘free’ (recalculated through mass balance modeling) concentrations for both parameters (Table S5). Figure 4 presents both the C_max_ and EC_50_ values across the cell types (panels A and C), along with the corresponding MOS (panels B and D) for either nominal or free concentrations (IC_10_ values are reported in Figure S3). Figure 4A shows that, when examining nominal concentrations of Cisplatin and Carboplatin, some cell types yield EC_50_ that are lower than human C_max_ values, indicating MOS < 1 for those cell types (Figure 4B). By contrast, compounds such as Dapagliflozin, Acarbose, and Adefovir demonstrated significantly higher MOS (exceeding 100). When free concentrations were used for comparisons (Figure 4C), both the human C_max_ values and the EC_50_ were shifted to the left. However, this shift was less pronounced or unchanged in some RPTEC media formulations, because cell type-specific formulations contain different levels of serum (0.2–10% by volume) that will result in different free drug fractions (Figure 4D). Consequently, this adjustment generally resulted in higher MOS values across the tested compounds.

**Figure 5:**
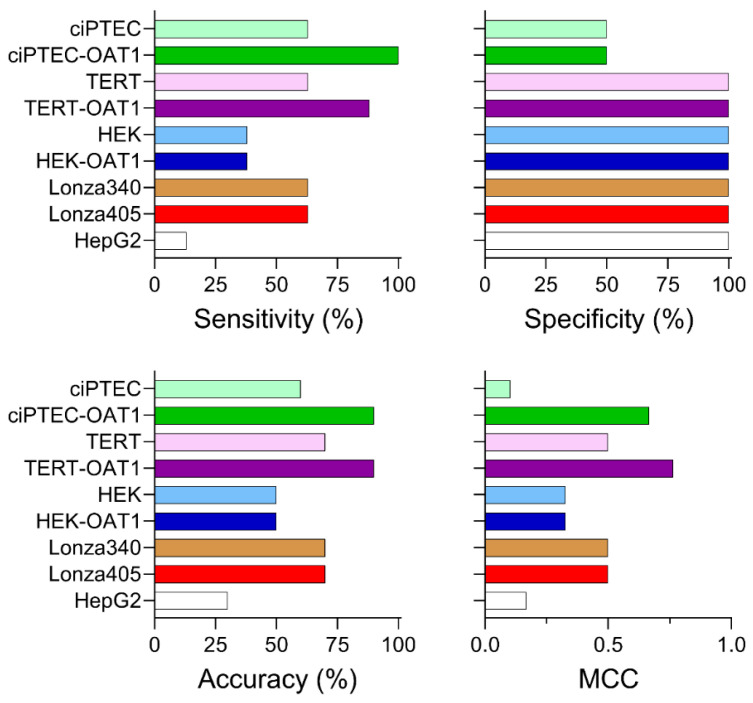
Nephrotoxicity prediction performance across tested cell sources based on EC_50_ values. Sensitivity, specificity, accuracy, and Matthew’s correlation coefficient (MCC) are reported for each cell source, based on a binary classification of compounds as nephrotoxic (positives) or non-nephrotoxic (negatives) in both in vivo and in vitro contexts.

To rank the cell types based on their ability to predict nephrotoxicity, sensitivity, specificity, accuracy, and Matthew’s correlation coefficient (MCC) were calculated based on EC50 values (Figure 5; prediction metrics based on IC10 are shown in Figure S4). For sensitivity, which measures the ability to correctly identify true positives, most RPTEC types (ciPTEC, TERT1, and both Lonza primary cell sources) achieved a sensitivity of 63%. By contrast, HEK cells (both basal and OAT1-overexpressing) showed a lower sensitivity of 38%, while HepG2 cells displayed the lowest sensitivity at 13%. Notably, OAT1 overexpression enhanced the sensitivity of TERT1 and ciPTEC lines to 88% and 100%, respectively. In terms of specificity, which reflects the ability to predict true negatives, all cell sources except for the ciPTEC lines demonstrated 100% specificity. The ciPTEC lines classified Ribavirin incorrectly as a nephrotoxic compound, though it is important to acknowledge that the small number of negative compounds (Ribavirin and Acarbose) is a limitation of the study. Regarding accuracy, which assesses the overall ability to correctly predict both true positives and negatives, ciPTEC and TERT parental and overexpressing lines showed similar predictive performances of 60–70%. The HEK cells achieved a 50% accuracy, while HepG2 cells were the least accurate at 30%. An overexpression of OAT1 again improved the accuracy in both ciPTEC and TERT1 lines, reaching 90% for both. Finally, the MCC values, which combine all predictive metrics to provide an overall ranking accounting for the imbalance in positives and negatives, placed the OAT1-overexpressing TERT1 and ciPTEC lines as the highest performers, with MCC scores of 0.764 and 0.667, respectively. Parental TERT1 and the two primary Lonza donors had moderate MCC scores of 0.5, while HepG2 and parental ciPTEC had the lowest scores (0.167 and 0.102, respectively). The low MCC for the ciPTEC parent line was primarily due to the false positive prediction of Ribavirin. Overall, ciPTEC and TERT1 lines emerged as the most predictive cell types, with OAT1 overexpression increasing the predictive performances of these cell sources.

## 4. Discussion

This study had the primary goal of comparing expression profiles and ability to predict nephrotoxicity among a broad panel of human RPTEC sources, including *OAT1*-overexpressing variants. About a third of all drugs are renally eliminated and of these, 92% are secreted [5]. Among uptake transporters, OAT1/3, and OCT2 have the highest number of interacting drugs and OAT1 shows the highest expression at both the mRNA and protein level [52]. Because human OAT1 and OAT3 have substantial overlap in their substrate specificity [53], which has also been observed across most basolateral and apical transporters [35], OAT1-overexpressing RPTECs have been a common in vitro model for studies of drug transport and toxicity. The need for such analysis is driven by the large number of options for cell-based RPTEC models that are available for end-users who need to be able to select the most “relevant” cell type for studies of drug or chemical screening. Seldom are such comparative analyses available from different vendors or in the published literature. This typically necessitates in-house small-scale comparative analyses—information that is not shared widely and leads to redundant studies and a lack of transparency. As new cell-based models become available, side-by-side comparisons with primary cells and among different vendors are especially important to establish proper contexts for drug screening and other applications [16]. Indeed, the challenge of comparing the predictivity of various cell types is well appreciated because of the differences in study design, concentrations, and drugs tested, as well as the phenotypes and parameters used for establishing the binary classification of nephrotoxicity [14]. In addition, as more complex in vitro systems, such as organoids and microphysiological systems, are becoming available for studies of kidney function and disease, cell type selection becomes a critical decision because the throughput of these models is rarely amenable to extensive comparisons of performance [54,55].

One path towards the wider adoption of in vitro models in toxicology in general, and in regulatory science in particular, is the availability of high-dimensional datasets, termed model-omics, on existing model systems, and storing them in publicly accessible databases [56]. Specifically, gene expression data are critical for defining the utility of the individual cell types as they allow for a thorough characterization of the basal transcriptomes in each cell type and across model systems. Most previous studies used limited gene sets probed using polymerase-chain reactions [29,30,31,32,34], and some recent publications used a larger targeted gene panel comprising ~1000 transcripts (the L1000 high-throughput transcriptomics platform) [27,28]. These studies of various in vitro models for nephrotoxicity used gene expression primarily in the context of treatment effects, either to determine the impact on known targets, or to discover new biomarkers. By contrast, our data allow for a comparison among cell sources before treatment and show that the cell source was the primary factor driving transcriptional differences, with cells first clustered by cell type, then by *OAT1* expression status. Indeed, the need to compare cell types across multiple vendor platforms, which may confer certain advantages and disadvantages, is a critical step in determining their potential regulatory and scientific utility [57,58]. While all types of data are useful, high-dimensional transcriptomics is especially informative because these data, when collected in concurrent experiments and on the same gene expression platform, can be continually mined in the future for the expression of target genes/pathways depending on the specific hypothesis or drug development needs, allowing various end-users to choose the most appropriate model to answer their own biological questions.

For example, only certain in vitro kidney models are known to retain functional transporter expression [59] and if *OAT1*-mediated drug transport is required, then using cell types that exhibit a high expression of OAT1 (or other transporters) may be important for informing the fit-for-purpose application of each cell type. In this regard, we observed that TERT1 and primary RPTECs from two donors clustered closely, whereas HEK and ciPTEC had more distinctive gene expression profiles. At the same time, we observed a marked co-induction of several transporters and other xenobiotic metabolism-related genes in *OAT1*-overexpressing cells. This may be the result of the phenomenon of the dynamic co-expression of functionally relevant metabolism genes in the kidney upon injury or drug treatment [60], or in this case due to supra-physiological levels of *OAT1* expression. This result is similar to our previous observation of a robust co-expression of other metabolism genes in TERT1-OAT1 cells [26]. Interestingly, the transcriptional effect of *OAT1* overexpression differed across three parental RPTEC types. We observed that *OAT1* overexpression exerted the highest effect in TERT1 cells (~150 DEGs), with ciPTEC in the middle (~50 DEGs) and HEK with very little effect (~25 DEGs). Indeed, the pathways impacted by *OAT1* expression varied widely, including metabolism and detoxification in HEK and ciPTEC cells, and immune response and cell cycle regulation in TERT cells. However, when kidney transporter/metabolism genes were selected for comparison among cell types, it was clear that while some genes are consistently expressed across all cell types (*MRP4*), many others were highly divergent between different sources. In general, among kidney cell sources, the HEK cell lines showed the lowest expression of these transport and metabolism genes.

Another determinant of the utility of in vitro models is their ability to correctly predict human toxicity within the preclinical stages of drug development [61]. Indeed, the primary goal of many published studies that tested different RPTECs was to evaluate their utility for the detection of renal tubular toxicity [14]. Typically, one or several cell sources are used, including primary RPTECs [27,28,29,30,34], stem cell-derived RPTEC [31], or immortalized cells and their transporter-overexpressing variants from a particular vendor [32,33]. Drug panels tested in these studies range from less than 10 to over 40 and most studies report the ability to classify compounds with ~80% accuracy using a range of readouts, from cell viability to gene expression or other molecular phenotypes. Most published studies, as noted above, used primary (freshly isolated or cryopreserved) RPTECs, cells that have a limited supply. Therefore, the information from these studies has primary importance for understanding the fundamental mechanisms of nephrotoxicity but may have limited value in terms of cross-lab/-study comparisons. The studies on immortalized cell lines and their transporter-overexpressing variants hold greater utility with respect to the potential use of these cells across a broader range of end-users (i.e., drug companies), but the publications that include these cells typically do not allow for a direct comparison among vendors and variants of the cell sources. Therefore, our study focused on the latter challenge—to provide a side-by-side comparison of drug effects on 8 different RPTECs and include a non-RPTEC type, HepG2 cells.

We found that the ciPTEC and TERT1 cells showed comparable sensitivity to known nephrotoxic agents, while the HEK cells were generally less sensitive. The sensitivity of the cryopreserved primary RPTECs was moderate, but not as high as many of the cell lines. HepG2 cells exhibited consistently lower sensitivity across all tested compounds compared to RPTEC lines, an observation that was reported in only one other published study on RPETC models for kidney toxicity applications [27]. Overall, these findings in parental cell lines confirm the utility of TERT1 and ciPTEC lines for nephrotoxicity assessments. We also found that *OAT1* overexpression increased RPTEC sensitivity to certain compounds, particularly Tenofovir and Adefovir and most notably in the ciPTEC and TERT1 lines. This effect is clearly driven by the fact that both compounds are OAT1 substrates [62]. In HEK293 cells, there was no significant difference in sensitivity between the parent and *OAT1*-overexpressing variants. These divergent results can be explained, at least in part, by the differences in the basal gene expression of *OAT1* among the three immortalized cell types. All *OAT1*-overexpressing variants exhibited a major upregulation of *OAT1* (*SLC22A6*), close to 1000-fold compared to their parental variants, but the background level of *OAT1* was the lowest in HEK cells. Indeed, the actions of multiple transporters are known to be responsible for the nephrotoxicity of Tenofovir and other tested drugs [63]. Therefore, the use of a single transporter-overexpressing variant may not fully recapitulate the in vivo pathways responsible for drug toxicity. Even a robust co-induction of various xenobiotic metabolism enzymes, which was observed in all three cell types, may be insufficient to equalize drug sensitivity among different immortalized RPTEC sources.

The quantitative comparisons of drug effects on different cell types also provide important information for future users. Specifically, the MOS analysis revealed differences based on cell type and compound, with some cell types showing EC_50_ values for Cisplatin and Carboplatin that were lower than human C_max_, resulting in MOS values below 1. By contrast, compounds like Dapagliflozin, Acarbose, and Adefovir had significantly higher MOS values, exceeding 100. Considering ‘free’ concentrations, both the C_max_ and EC_50_ values shifted to the left, especially for drugs with known serum-binding effects. This shift resulted in generally higher MOS values for the tested compounds, though the extent varied based on media formulations for specific RPTECs that require very different FBS levels. The differences in the MOS for the ‘free’ concentrations suggest that serum binding needs to be carefully considered in future studies and cell type selection because reduced C_max,free_ values can require lower exposure concentrations in vitro depending on the serum/protein content in the culture media. Additionally, it is also possible that the in vivo kidney concentration may be higher for some compounds compared to their plasma C_max._

While risk characterization using the MOS approach is informative for specific compounds, we also calculated traditional classification accuracy metrics using data from across all compounds. We found that the sensitivity of the predictions of nephrotoxic compounds (63%) was identical for the parental immortalized RPTECs ciPTEC and TERT1, as well as both primary RPTEC lines, and showed the same ability to predict true positives (63%). *OAT1*-overexpressing variants of ciPTEC and TERT1 lines were also able to correctly categorize Dapagliflozin and Tenofovir. While Tenofovir is an OAT1 substrate [62], OAT1 plays no role in Dapagliflozin pharmacokinetics [64] because it is a P-gp substrate [65] and an SGLT2 (SLC5A2) inhibitor [66]. ciPTEC-OAT1 cells were also able to correctly predict Adefovir as a toxic compound. While TERT1-OAT1 cells did respond to Adefovir, their viability did not fall below 50% at the highest tested concentration (300 μM); therefore, an EC_50_ could not be derived. Adefovir is known to exert renal toxicity through an increase in intracellular influx through an OAT1-controlled mechanism [67] and cell line-dependent sensitivity to its effects is an important consideration for the future selection of cell types for screening. While ciPTEC-OAT1 cells showed 100% sensitivity (compared to 88% for TERT-OAT1), the opposite was true for specificity (Ribavirin was classified incorrectly by ciPTEC-OAT1), yielding a comparable overall accuracy for the two cell types. Overall, our data demonstrate that both ciPTEC-OAT1 and TERT-OAT1 cell types provide the highest predictive ability, as demonstrated by their higher MCC scores compared to other cell sources. Given the ease of use of these cell lines over primary cultures, they are particularly advantageous as preliminary screening tools for identifying nephrotoxic risk. These models could be especially valuable for targets with a priori risk for tubular injury, offering reliable predictions before advancing to more complex in vivo studies. It is also important to note that HepG2 cells, which are commonly employed for routine cytotoxicity testing in the pharmaceutical industry, may not be effective for detecting nephrotoxicants. This finding challenges the conclusions of Lin and Will [68] who suggested the broad utility of HepG2 cells across organ-specific toxicity assessments. Our results underscore the necessity of using kidney-specific cell models like ciPTEC-OAT1 and TERT-OAT1 for accurate nephrotoxicity evaluation, highlighting their critical role in refining drug development pipelines.

It is important to acknowledge several limitations of this study. First, we tested eight RPTEC sources but only two primary lines were included. Significant inter-individual differences in renal clearance likely exist among humans, similar to the large inter-species variations observed [69]. However, most of the known human variability has been attributed to pre-existing kidney disease [2]. Although OAT1 is one of the most abundant renal transporters in human kidneys, it exhibits relatively low variability [70]. Further studies are needed to assess the impact of inter-individual variability in transporters and how it may be represented in future in vitro models for nephrotoxicity research. Second, these studies were performed in static cultures and some studies have demonstrated that media flow and shear stress may improve the function and physiological relevance of RPTECs in culture [71,72,73]. While studies of cell source comparisons are possible under flow, static cultures could aid in the selection of cell types because of the relatively low throughput and high cost of microfluidics-based devices. Third, our study used a toxicology-relevant, but still limited, gene expression panel. The TempO-seq S1500+ panel was selected to be representative of most human tissues, similar to the design of the L1000 targeted high-throughput transcriptome method [38]. In addition, S1500+ also is over-represented for xenobiotic metabolism genes and other toxicologically relevant transcripts and it has been shown that the results from the S1500+ platform are consistent with findings on other genome-wide transcriptome methods [74]. However, because it represents only about 10% of the total transcriptome, the utility of our gene expression data with respect to model-omics information [56] may be limited. Finally, we acknowledge that the drug panel tested herein, 12 compounds, is not as extensive as that used in several recent publications. However, because the focus was on the comparison of cell types, not drugs, we reason that this limitation can be addressed in future research by focusing on a smaller number of the most relevant cells.

## 5. Conclusions

In conclusion, this study’s comparative evaluation of various renal proximal tubule epithelial cell sources provides critical insights for end users aiming to identify the most suitable cell type for their screening studies. By offering a comprehensive analysis of gene expression profiles, a comparative analysis of drug effects, and nephrotoxicity prediction performance evaluation, this study equips users with data-driven guidance to select cell models that align with their specific research goals, ensuring both accuracy and relevance in preclinical nephrotoxicity assessments. Specifically, this study strengthens the fact that improved cell options for renal proximal tubule are needed but shows that *OAT1*-overexpressing RPTEC are a superior model compared to the background cell type.

## Figures and Tables

**Table 1:** Drugs tested in this study.

Common Drug Name	Chemspyder ID	Formula	Human Serum C_max_ (μM) *	Human Nephro-Toxicity Risk ^#^	Concentrations Tested (μM)	Tested Range of C_max_	Vehicle
Cisplatin	76401	Pt(NH_3_)_2_Cl_2_	30	High	0.3, 1, 3, 10, 30, 100, 300	0.001–10	DMSO (0.5%)
Carboplatin	8514637	C_6_H_12_N_2_O_4_Pt	24–149	Intermediate		0.01–2	DMSO (0.5%)
Canagliflozin	26333259	C_24_H_25_FO_5_S	2.75–10.5	High		0.1–30	DMSO (0.5%)
Dapagliflozin	8063384	C_21_H_25_ClO_6_	0.176–0.41	Intermediate		2–700	DMSO (0.5%)
Gentamicin	390067	C_21_H_43_N_5_O_7_	41.2	High		0.01–7	Media
Streptomycin	18508	C_21_H_39_N_7_O_12_	73.2	Intermediate		0.01–4	DMSO (0.5%)
Tenofovir	408154	C_9_H_14_N_5_O_4_P	1.1	High		0.3–300	DMSO (0.5%)
Adefovir	54252	C_8_H_12_N_5_O_4_P	0.07	High		4–4000	DMSO (0.5%)
Telithromycin	2273373	C_43_H_65_N_5_O_10_	2.5	High		0.1–120	DMSO (0.5%)
Rifampicin	10468813	C_43_H_58_N_4_O_12_	10–27	High		0.03–30	DMSO (0.5%)
Acarbose	392239	C_25_H_43_NO_18_	0.008–0.05	Low		37–6000	DMSO (0.5%)
Ribavirin	34439	C_8_H_12_N_4_O_5_	2.6–5.2	Low		0.1–60	DMSO (0.5%)

* See Table S2 for the sources for information on human serum C_max_ values. ^#^ Human Nephrotoxicity Risk for each drug was defined in [30,33]. Compounds with high and intermediate risk were considered as positives and those with low risk as negatives for cell performance evaluation.

**Table 2:** Gene Ontology (GO) pathways and overlapping DEGs for cell type-specific clusters shown in Figure 1.

GO Pathway Name	Overlapping Genes	# in Pathway	# Over-Lapping	p_adj_
Primary (Lonza 340 and 405) RPTECs				
Drug Metabolic Process	*ADH1A*, *CBR1*, *CYP1A1*, *CYP2C9*, *CYP3A4*, *CYP3A5*, *CYP4F2*, *EPHX2*, *FMO5*, *UGT1A10*, *UGT1A3*, *UGT1A4*, *UGT1A6*, *UGT1A7*, *UGT1A8*, *UGT1A9*	27	16	1.3 × 10^−10^
Response to Metal Ion	*AKR1C3*, *ALB*, *AQP1*, *AQP9*, *C3*, *CA2*, *CD14*, *CLU*, *CYP1A1*, *FGB*, *FGG*, *FN1*, *HIF1A*, *HMGCS2*, *HMOX1*, *ICAM1*, *MAOB*, *MT1E*, *MT1F*, *MT1G*, *MT1M*, *MT1X*, *MT2A*, *NDRG1*, *NFATC4*, *NPC1*, *PTK2B*, *SLC18A2*, *VCAM1*	132	29	1.1 × 10^−7^
Organic Anion Transport	*ABCC2*, *ABCC4*, *ACE2*, *ACSL1*, *ALB*, *APOA1*, *APOC1*, *APOE*, *AQP1*, *AQP9*, *CA2*, *CA9*, *FABP1*, *NR1H4*, *PLIN2*, *SCARB1*, *SLC10A2*, *SLC2A2*, *SLC2A3*, *SLC51B*, *SLC7A7*, *SLCO2B1*	82	22	2.2 × 10^−7^
Innate Immune Response	*C1R*, *C1RL*, *C1S*, *C3*, *CD14*, *CLU*, *FGB*, *GBP5*, *HLA-C*, *HLA-DQB1*, *HLA-DRA*, *HLA-DRB5*, *HLA-E*, *ICAM1*, *IFI27*, *IFITM2*, *IFITM3*, *IKBKE*, *IRF8*, *MT2A*, *NR1H4*, *PTK2B*, *TRIM22*, *VCAM1*, *XAF1*	170	25	6.5 × 10^−4^
Extracellular Structure Organization	*A2M*, *CTSG*, *DCN*, *FBN1*, *FGB*, *FGG*, *FN1*, *HPN*, *ICAM1*, *ITGB5*, *LUM*, *MMP1*, *SPP1*, *TTR*, *VCAM1*	98	15	7.7 × 10^−3^
TERT1 and TERT1-OAT1 RPTECs				
Immune Response	*B2M*, *C1QB*, *CCL2*, *CD40*, *CD44*, *CTSS*, *CXCL1*, *CXCL2*, *CXCL8*, *EDN1*, *ETS1*, *FYB*, *GCNT3*, *HIST1H2BE*, *HIST1H2BI*, *HLA-A*, *HLA-B*, *HLA-DMB*, *HLA-F*, *IFI30*, *IFIT3*, *IFITM1*, *IL18*, *IL4R*, *IL6*, *IRF7*, *KDM5D*, *LCK*, *LCN2*, *LTF*, *MALT1*, *MX1*, *NCF2*, *NOTCH1*, *OAS1*, *PPBP*, *RELB*, *S100A13*, *SAA1*, *SBSPON*, *THBS1*, *TNF*, *TNFRSF21*, *TNFSF12*, *TNFSF13*, *TNFSF13B*	280	46	3.5 × 10^−4^
Positive Regulation of Ion Transport	*ATP1B1*, *B2M*, *BAK1*, *BMP4*, *CALM3*, *CAV1*, *CCL2*, *CTSS*, *EDN1*, *GLRX*, *MYLK*, *NOS1*, *PDGFB*, *STC1*, *TRPC6*	59	15	2.8 × 10^−3^
Regulation of Cell Differentiation	*ADGRG1*, *AREG*, *B2M*, *BMP4*, *CAV1*, *CCND1*, *CYR61*, *DUSP6*, *EDN1*, *EREG*, *ETS1*, *GDF15*, *HLA-B*, *IFITM1*, *IGFBP3*, *IL18*, *IL4R*, *IL6*, *INSIG1*, *IRF7*, *KLF5*, *L1CAM*, *LTF*, *MAFF*, *NANOG*, *NFKBIA*, *NOS1*, *NOTCH1*, *NRP1*, *PAX8*, *PDGFB*, *PRKX*, *SEMA4B*, *SFN*, *SNAP25*, *SOCS3*, *SOX2*, *SPDEF*, *STAT3*, *TGFBR2*, *TIMP2*, *TNF*, *TNFRSF21*, *TRPC6*, *WNT7B*	323	45	7.6 × 10^−3^
HEK and HEK-OAT1 RPTECs				
Cell Development	*ACTN2*, *ADCY1*, *BMP6*, *BTG2*, *CEBPA*, *CHN1*, *CXCR4*, *EOMES*, *EPHA3*, *FBXO5*, *FGFR2*, *FLRT2*, *FOXA1*, *FYN*, *GABRB3*, *GAP43*, *GATA2*, *GATA6*, *GDF7*, *GSTM3*, *HIST1H2BA*, *HOXD10*, *ID2*, *KIF5C*, *KIT*, *LEF1*, *LMOD1*, *MAPT*, *PAX6*, *PHGDH*, *PODXL*, *PRKCQ*, *PRKG1*, *S100B*, *SLC1A3*, *SULF1*, *TFAP2A*, *TIAM1*, *WASF3*, *WT1*, *ZP3*	289	41	5.4 × 10^−4^
Cellular Amino Acid Biosynthesis	*ASS1*, *BCAT1*, *FOLH1*, *GAD1*, *GLUL*, *PHGDH*, *PSAT1*, *SLC1A3*	27	8	9.4 × 10^−3^
Mitotic Cell Cycle	*BCAT1*, *BTG2*, *CDC25A*, *CDC45*, *CDC6*, *CDC7*, *CDCA5*, *CDKN2A*, *CDT1*, *CHEK2*, *DLGAP5*, *E2F7*, *ESPL1*, *FANCI*, *FBXO5*, *FSD1*, *ID2*, *KIF11*, *KIF4A*, *MCM10*, *MCM7*, *MDM2*, *MYBL2*, *NCAPD2*, *OIP5*, *ORC1*, *PAX6*, *PCNA*, *PSRC1*, *RRM2*, *TIMELESS*, *TTK*, *ZWINT*	260	33	9.4 × 10^−3^
ciPTEC and ciPTEC-OAT1 RPTECs				
Extracellular Structure Organiz.	*CCDC80*, *COL12A1*, *COL16A1*, *COL3A1*, *COL5A1*, *COL6A3*, *CTGF*, *ENG*, *LOXL1*, *MMP2*, *MMP3*, *PECAM1*, *PRSS2*, *SERPINE1*	98	14	4.1 × 10^−4^
Regulation of Acute Inflamm. Response	*C3AR1*, *CFH*, *FCER1G*, *IL1B*, *PLA2G4A*, *PTGS2*	21	6	2.8 × 10^−3^
Regulation of Extrinsic Apoptosis	*G0S2*, *GDNF*, *IL1A*, *IL1B*, *INHBA*, *SERPINE1*, *SNAI2*, *SRPX*, *TERT*	64	9	7.1 × 10^−3^

## Data Availability

All raw data files can be found at the following links hosted by Eve Analytics: https://eve.eveanalytics.com/assays/assaystudy/1317/; https://eve.eveanalytics.com/assays/assaystudy/1324/; https://eve.eveanalytics.com/assays/assaystudy/1329/, accessed on 17 February 2025.

## Supplementary Materials

The following supporting information can be downloaded at: https://www.mdpi.com/article/10.3390/biomedicines13030563/s1, Figure S1: Viability response of TERT1-Parent and TERT1-OAT1 RPTEC lines after 72 h exposure to Gentamicin and Streptomycin; Figure S2: Heatmap displaying IC10 values derived from cell viability data after a 72 h exposure; Figure S3: IC_10_ values vs. human Cmax and margins of safety (MOS) across cell sources; Figure S4: Nephrotoxicity prediction performance across tested cell sources based on IC_10_ values. Table S1: IC10 and EC50 PODs derived from cell viability data after 72 h drug exposure; Table S2: Human Cmax ranges for exposure compounds; Table S3: Chemical-related parameters for in vitro Mass Balance Model; Table S4: System-related Parameters for in vitro Mass Balance Model; Table S5: Nominal and calculated “Free” concentrations for human Cmax used in margin of safety (MOS) calculations. File S1: DEGs OAT1 vs. Parent; File S2: GOBP OAT1 vs. Parent; File S3: Heatmap Union DEGs.

## Abbreviations

The following abbreviations are used in this manuscript:

RPTEC	Renal Proximal Tubule Epithelial Cells
TERT1	Telomerase Reverse Transcriptase
ciPTEC	Conditionally Immortalized Proximal Tubule Epithelial Cells
HEK	Human Embryonic Kidney
OAT1	Organic Anion Transporter 1
DMSO	Dimethyl Sulfoxide
EGF	Epithelial Growth Factor
FBS	Fetal Bovine Serum
DMEM	Dulbecco’s Modified Eagle Medium
ATP	Adenosine triphosphate
PCA	Principal Component Analysis
DEGs	Differentially Expressed Genes
POD	Point of Departure
Fub	Unbound Fraction
IVMBM	In vitro Mass Balance Model
Kow	Octanol–Water Partitioning Coefficient
Kaw	Air–water Partitioning Coefficient
Koa	Octanol–Air Partitioning Coefficient
EAS-E	Exposure And Safety Estimation
MCC	Matthew’s correlation coefficient
MOS	Margin of Safety
