# Supplementary material for: Comparative Analysis of Proximal Tubule Cell Sources for In Vitro Studies of Renal Proximal Tubule Toxicity
Source: Biomedicines. 2025 Feb 24;13(3):563. doi: 10.3390/biomedicines13030563 (PMC11940618; doi:10.3390/biomedicines13030563)

Supplemental Materials:

Table S1. IC_{10} and EC_{50} PODs derived from cell viability data after 72h drug exposure.

IC_{10}	ciPTEC -OAT1	ciPTEC	TERT1-OAT1	TERT1	HEK293-OAT1	HEK293	Lonza (#340)	Lonza (#405)	HepG2
*Cisplatin	9.0	8.8	12.6	32.8	106	56.0	53.7	33.2	0.4
Carboplatin	79.8	50.3	89.3	222	300	248	99.8	202	27.9
*Canagliflozin	1.6	3.6	22.6	28.0	10.0	5.4	23.9	51.2	27.0
Dapagliflozin	70.7	10.4	79.6	57.1	12.5	3.9	300	300	300
*Gentamicin	300	300	300	300	142	13.6	300	300	82.0
Streptomycin	300	300	300	300	300	300	300	300	300
*Tenofovir	30.7	300	41.1	257	300	300	202	300	300
*Adefovir	3.4	300	1.0	300	300	300	300	300	300
*Telithromycin	62.8	61.1	191	93.3	24.8	21.4	82.9	137	12.7
*Rifampicin	9.5	61.3	23.9	36.9	83.0	75.8	209	108	96.5
Acarbose	300	300	300	300	300	5.9	300	209	300
Ribavirin	24.9	22.1	3.9	83.0	300	300	71.0	102	25.2

EC_{50}	ciPTEC-OAT1	ciPTEC	TERT1-OAT1	TERT1	HEK293-OAT1	HEK293	Lonza (#340)	Lonza (#405)	HepG2
*Cisplatin	11.6	12.8	69.0	40.9	300	300	123	248	300
Carboplatin	99.1	81.4	216	272	300	300	237	265	300
*Canagliflozin	43.1	59.4	40.7	70.7	37.4	32.7	42.5	63.4	78.8
Dapagliflozin	202	300	201	300	300	300	300	300	300
*Gentamicin	300	300	300	300	300	300	300	300	300
Streptomycin	300	300	300	300	300	300	300	300	300
*Tenofovir	69.0	300	175	300	300	300	300	300	300
*Adefovir	39.5	300	300	300	300	300	300	300	300
*Telithromycin	165	226	244	170	57.5	56.6	165	274	300
*Rifampicin	96.9	179	131	172	166	156	299	214	300
Acarbose	300	300	300	300	300	300	300	300	300
Ribavirin	170	228	300	300	300	300	300	300	300

Table S2. Human C_{max} ranges for exposure compounds

Drug name	CAS Number	Dose	Cmax (mg/L)	Ref
Cisplatin	15663-27-1	One Hour Infusion Group	5.37	Rajkumar et al., 2016
		Three Hour Infusion group	9.03	Rajkumar et al., 2016
		2-500 uM	6.17	Sjögren et al., 2018
Carboplatin	41575-94-4	75 mg/m2	9.06	Oguri et al., 1988
		150 mg/m2	14.09	Oguri et al., 1988
		300 mg/m2	31.59	Oguri et al., 1988
		375 mg/m2	38.99	Oguri et al., 1988
		450 mg/m2	55.39	Oguri et al., 1988
Canagliflozin	842133-18-0	100mg	1.178	Chen et al., 2015
		300mg	4.113	Chen et al., 2015
		50mg	0.426	Devineni et al., 2013
		100mg	1.096	Devineni et al., 2013
Dapagliflozin	461432-26-8	10mg	0.08547	Oroian et al., 2020
		10mg	0.158	EC
Gentamicin	1405-41-0	7.4 (6.7-8.1)	14.5-20.6	Gonçalves-Pereira et al., 2010
		6-1500uM	0.89789	Sjögren et al., 2018
		6.6 ± 2.3 mg/kg	15.7	Roger et al., 2015
Streptomycin	3810-74-0	18 (10-23): Intramuscular; Unit: mg/kg	2.9-85.2	Zhu et al., 2012
		14 (11-25): Intravenous; Unit: mg/kg	9-107	Zhu et al., 2012
Tenofovir	147127-20-6	300mg	0.2222	Brooks et al., 2019
		300mg	1.77	Blum et al., 2013
		300mg	0.293	Chittick et al., 2006
		300mg	0.36	Droste et al., 2004
		0.7-167uM	0.3274194	Sjögren et al., 2018
Adefovir	106941-25-7	10mg	0.0193	Kearney et al., 2013
		10mg	0.0149	Bi et al., 2005
		20mg	0.03	Bi et al., 2005
		167uM	0.01830373	Sjögren et al., 2018
Telithromycin	191114-48-4	400mg (a single oral dose)	0.8	Namour et al., 2001
		800mg (a single oral dose)	1.9	Namour et al., 2001
		1200mg (a single oral dose)	4.07	Namour et al., 2001
		400mg (7 days of once-daily oral dosing)	0.829	Namour et al., 2001
		800mg (7 days of once-daily oral dosing)	2.27	Namour et al., 2001
		1200mg (7 days of once-daily oral dosing)	4.48	Namour et al., 2001
Rifampicin	13292-46-1	450mg (patient)	0.0066	Rafiq et al., 2010
		450mg (health)	0.005518	Rafiq et al., 2010
		single-dose	8.98	Stott et al., 2018
		steady-state dosing	5.79	Stott et al., 2018
		750mg	6.1-22.2	Yunivita et al., 2016
		900mg	5.7-28.3	Yunivita et al., 2016
		6-1500uM	12.014924	Sjögren et al., 2018
Acarbose	56180-94-0	300ug	0.00528-0.0681	Fernandez et al., 2019
		600ug	0.0115-0.0368	Fernandez et al., 2019
		0.7-167uM	0.0497112	Sjögren et al., 2018
Ribavirin	36791-04-5	600mg	0.643	Martin and Jensen, 2008

Table S3. Chemical-related parameters for *in vitro* Mass Balance Model

Drug name	CAS Number	MW (g/mol)	MP (oC)	log KOW,N	log KAW,N	CSAT,W,N (mg/L)	Source
**Cisplatin**	15663-27-1	298.03	270	-2.19	-30	2530	PMID: 25058905
**Carboplatin**	41575-94-4	371.25	230	-2.3	-30	14000	Pubchem, PMID 10732773
**Canagliflozin**	842133-18-0	444.52	297	4.19	-19.3	68.45608	Eas-e-suite database
**Dapagliflozin**	461432-26-8	408.88	65	2.7	-30	1600	Pubchem, CDER document 209091Orig1s000
**Gentamicin**	1405-41-0	477.6	309	-2.14	-30.6	606552	Eas-e-suite database
**Streptomycin**	3810-74-0	581.57	466	-5.51	-40.1	1134061.5	Eas-e-suite database
**Tenofovir**	147127-20-6	287.21	163	-2.16	-20.7	338907.8	Eas-e-suite database
**Adefovir**	106941-25-7	273.19	161	-2.68	-21	483546.3	Eas-e-suite database
**Telithromycin**	191114-48-4	812.018	188	3	-30	300	Pubchem
**Rifampicin**	13292-46-1	822.94	494	3.1	-43.8	1398.998	Eas-e-suite database
**Acarbose**	56180-94-0	645.6	462	-7.68	-46.6	1149168	Eas-e-suite database
**Ribavirin**	36791-04-5	244.2	229	-1.85	-23.6	141880.2	Eas-e-suite database

Table S4. System-related Parameters for *in vitro* Mass Balance Model

Cell type	Test System	Cell number	% of Serum
**ciPTEC-OAT1**	96 well; Flat bottom	1E-30 (No cell system)	10.00%
**ciPTEC-Parent**	96 well; Flat bottom	1E-30 (No cell system)	10.00%
**HEK-OAT1**	96 well; Flat bottom	1E-30 (No cell system)	10.00%
**HEK-Parent**	96 well; Flat bottom	1E-30 (No cell system)	10.00%
**HepG2**	96 well; Flat bottom	1E-30 (No cell system)	10.00%
**Lonza_340**	96 well; Flat bottom	1E-30 (No cell system)	0.50%
**Lonza_405**	96 well; Flat bottom	1E-30 (No cell system)	0.50%
**TERT-OAT1**	96 well; Flat bottom	1E-30 (No cell system)	0.20%
**TERT-Parent**	96 well; Flat bottom	1E-30 (No cell system)	0.20%

Table S5. Nominal and calculated "Free" concentrations for human C_{max} used in margin of safety (MOS) calculations.

Chemical Name	Total Cmax_max (uM)	Free Cmax_max (uM)
Cisplatin	30.30	27.27
Carboplatin	149.20	149.20
Canagliflozin	9.25	0.09
Dapagliflozin	0.39	0.03
Tenofovir	6.16	5.73
Adefovir	0.11	0.11
Telithromycin	5.52	2.26
Rifampicin	34.39	6.88
Acarbose	0.11	0.08
Ribavirin	2.63	2.63

Figure S1. Viability response of TERT1-Parent and TERT1-OAT1 RPTEC lines after 72-hour exposure to Gentamicin and Streptomycin. Dose-response curves show the viability following a 72-hour exposure. The IC_{10} and EC_{50} values are indicated for each condition, and individual data points are plotted along with a fitted line (n≥3).

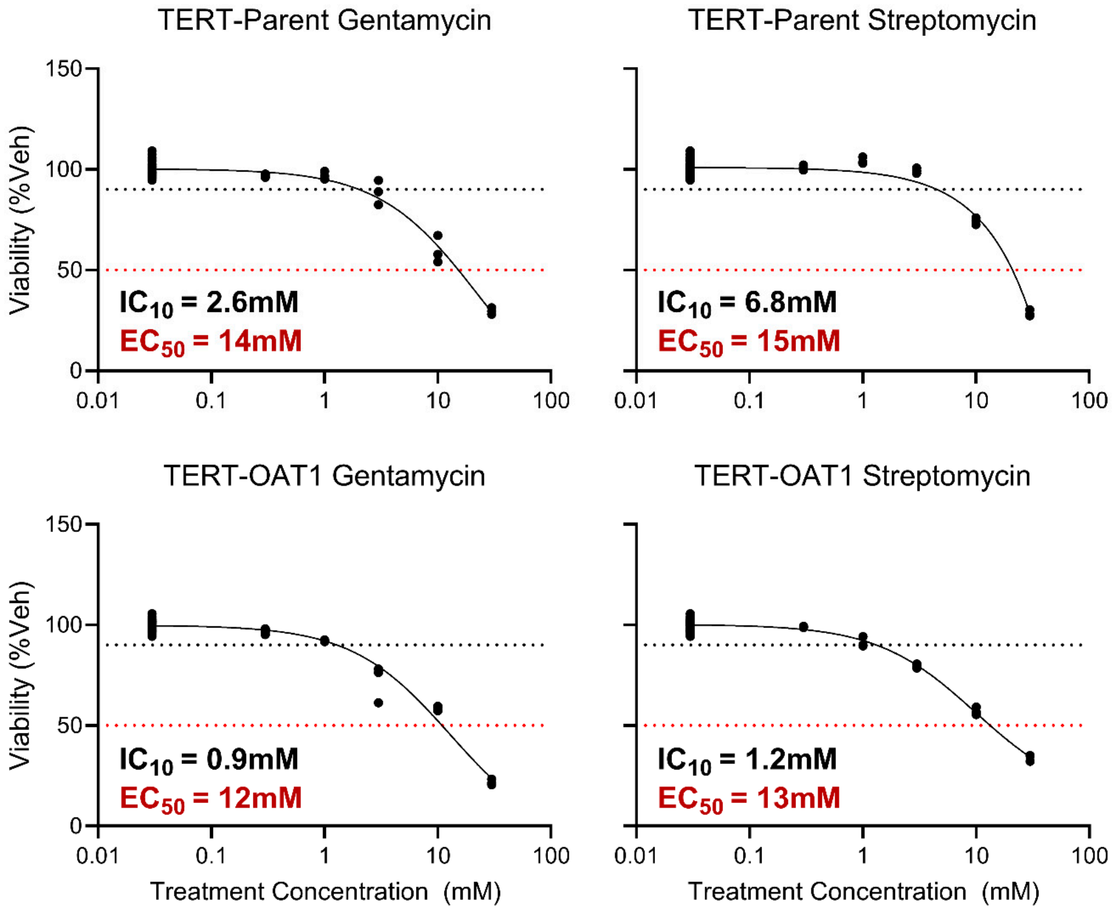

Figure S2. Heatmap displaying IC_{10} values derived from cell viability data after a 72h exposure.

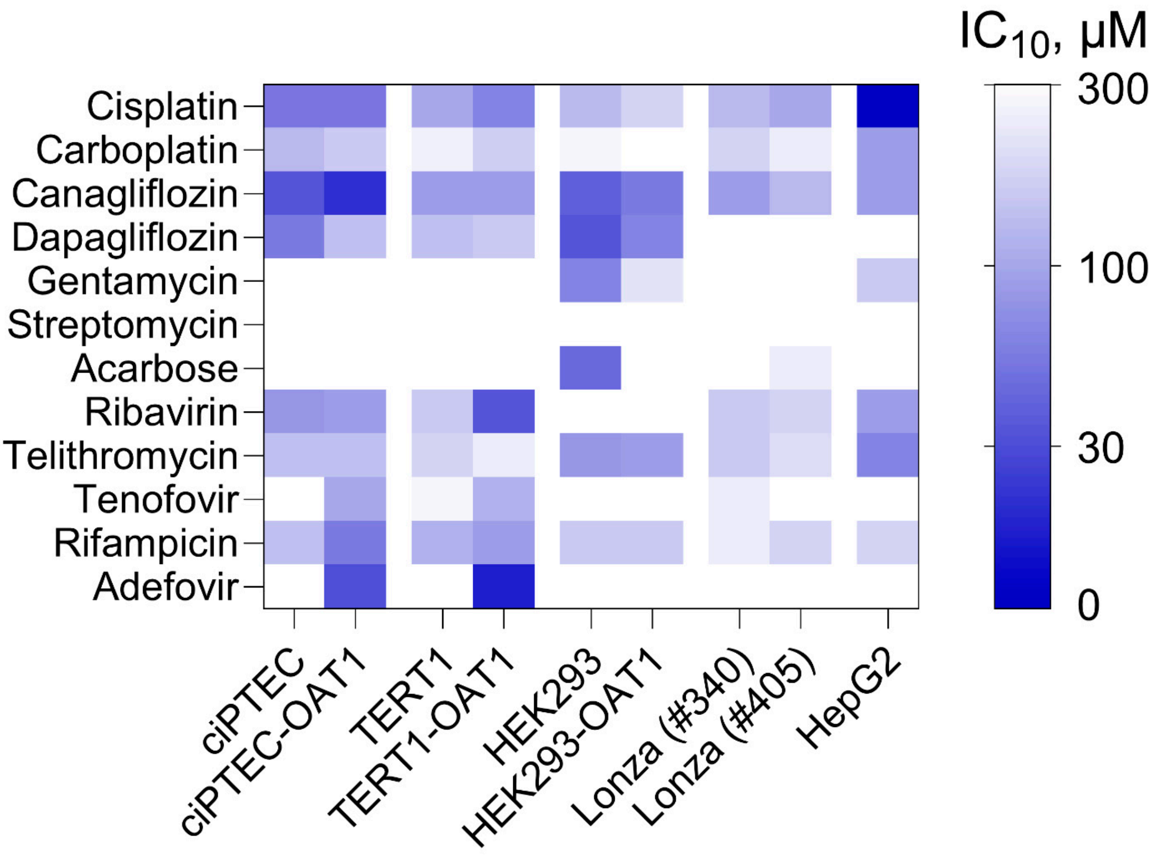

Figure S3. IC_{10} values vs. human Cmax and margins of safety (MOS) across cell sources. (A) Experimental IC_{10} values for each cell type plotted against reported human C_{max}. (B) MOS calculated using the ratio of human C_{max} to IC_{10}. (C) $IC_{10,free}$ values (free concentrations in media) plotted against calculated $C_{max,free}$ in human serum, using mass balance modeling. (D) MOS calculated using the ratio of $C_{max,free}$ to $IC_{10,free}$, accounting for free concentrations in media and serum.

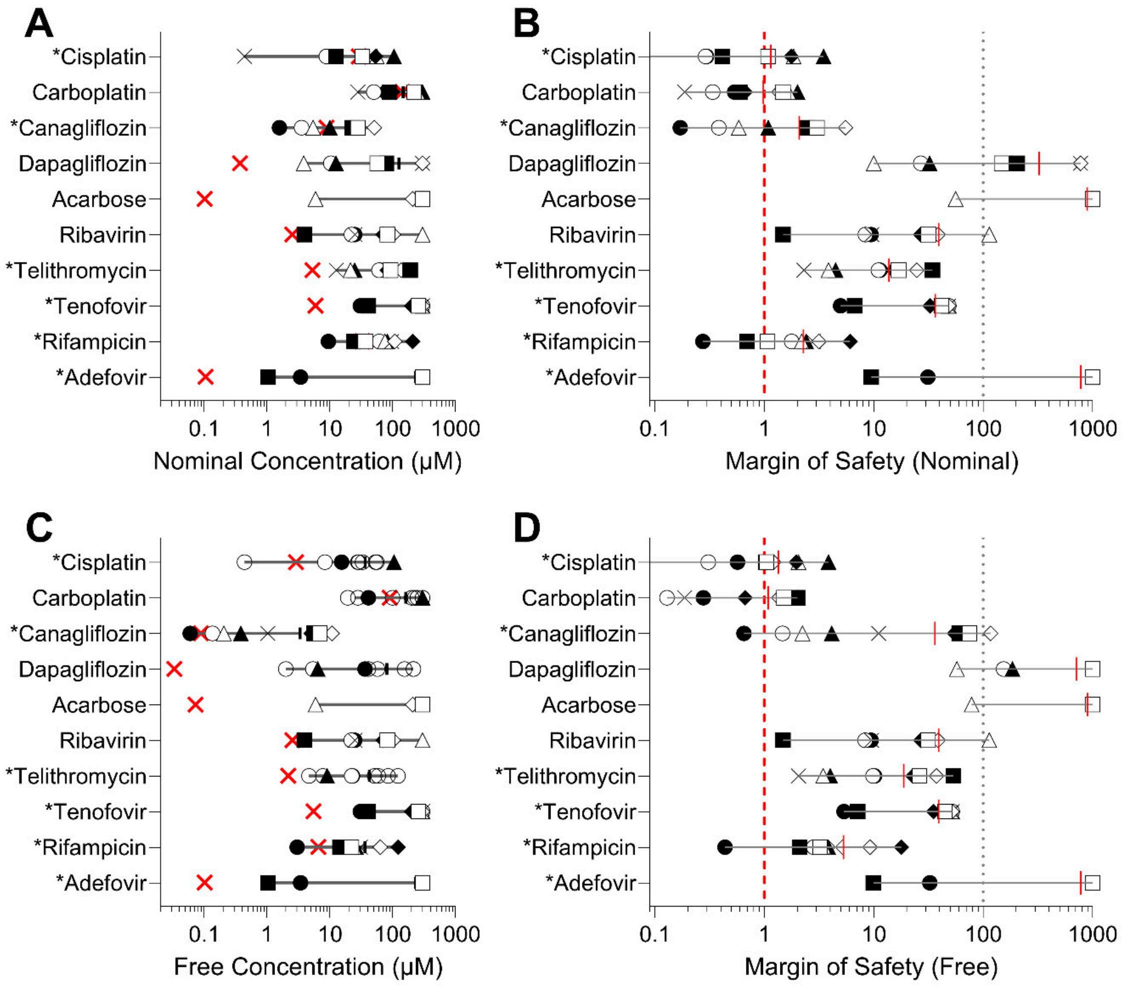

Figure S4. Nephrotoxicity prediction performance across tested cell sources based on IC_{10} values. Sensitivity, specificity, accuracy, and Matthews correlation coefficient (MCC) are reported for each cell source, based on binary classification of compounds as nephrotoxic (positives) or non-nephrotoxic (negatives) in both *in vivo* and *in vitro* contexts.

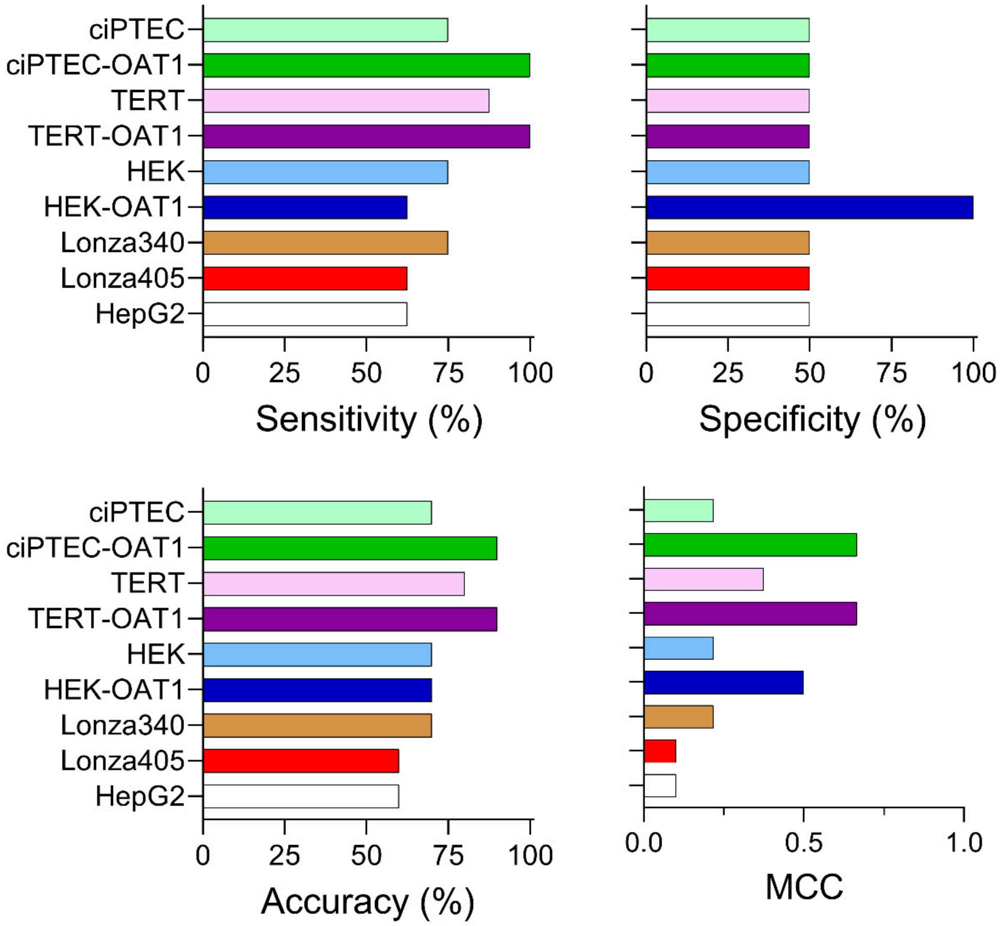

Supplement: Supplementary file 1 [file biomedicines-13-00563-s001.zip › Sakolish et al Supplemental Tables and Figures.pdf]
